# Lack of Mimivirus Detection in Patients with Respiratory Disease, China

**DOI:** 10.3201/eid2211.160687

**Published:** 2016-11

**Authors:** Xiao-Ai Zhang, Teng Zhu, Pan-He Zhang, Hao Li, Yan Li, En-Mei Liu, Wei Liu, Wu-Chun Cao

**Affiliations:** State Key Laboratory of Pathogen and Biosecurity, Beijing Institute of Microbiology and Epidemiology, Beijing, China (X.-A. Zhang, T. Zhu, P.-H. Zhang, H. Li, W. Liu, W.-C. Cao);; Graduate School of Anhui Medical University, Hefei, China (T. Zhu);; 307 Hospital, Beijing (Y. Li);; Children’s Hospital of Chongqing Medical University,; Chongqing, China (E.-M. Liu)

**Keywords:** mimivirus, viruses, pneumonia, epidemiology, respiratory disease, respiratory infections, China

**To the Editor:** Mimivirus (*Acanthamoeba polyphaga* mimivirus), which was initially identified as a gram-positive parasitic bacterium, is the first member of the virus family Mimiviridae (*1*,*2*). Although mimivirus was initially isolated in the context of a human pneumonia outbreak, its pathogenicity to humans remains uncertain.

Mimivirus DNA was detected in a bronchoalveolar lavage specimen from a 60-year-old comatose patient with hospital-acquired pneumonia (*3*) and isolated from a 72-year-old woman with pneumonia (*4*). However, many studies that used a PCR detection method reported that mimivirus is a negligible cause of respiratory infections in humans (*5*–*9*). Furthermore, serologic evidence for this new virus was suggested as being caused by cross-reactivity to *Francisella tularensis* (*10*). To estimate the prevalence of mimivirus and its potential role in causing respiratory infections, we conducted a retrospective study by screening 2 cohorts of patients in China for this virus.

Cohort 1 was composed of 2,304 children with acute lower respiratory tract infections who were hospitalized in Children’s Hospital of Chongqing Medical University (Chongqing, China), from whom nasopharyngeal aspirates were obtained during June 2011–July 2015. Patients ranged in age from 1 month to 16 years (median 15.0 months), and 2,034 (88.3%) had pneumonia.

Cohort 2 was composed of 768 children (43 hospitalized patients and 725 outpatients) and 624 adults (440 hospitalized patients and 184 outpatients) with acute lower or upper respiratory tract infections at 307 Hospital (Beijing, China), from whom throat swab specimens were obtained during January 2013–December 2015. Children ranged in age from 1 to 192 months (median 48.0 months), and 62 (8.1%) had pneumonia. Adults ranged in age from 17 to 95 years (median 52.9 years) and 401 (55.3%) had pneumonia.

Virus nucleic acids were extracted by using a QIAamp MinElute Virus Spin Kit (QIAGEN, Hilden, Germany). Mimivirus was detected by real-time reverse transcription PCR (RT-PCR) as described (*4*).

Of the 3,696 patients, only 1, a 6-month-old boy, had a positive real-time RT-PCR result (cycle threshold [C_t_] 31) for mimivirus. This positive result was verified by using 2 other RT-PCRs specific for the helicase and thiol oxidoreductase genes and a nested RT-PCR (*3**,**6*). The PCR specific for thiol oxidoreductase showed a positive result (C_t_ 36). An independent retesting that was performed on this positive sample in the laboratory affiliated to PLA 307 hospital was also positive. However, we could not amplify mimivirus sequences by using nested RT-PCR.

Samples from 3,696 patients were simultaneously screened for influenza virus; respiratory syncytial virus; parainfluenza virus types 1, 2, 3, and 4; metapneumovirus; human rhinovirus; human adenovirus; coronavirus, and human bocavirus by using PCR. All 11 of these respiratory viruses were detected at prevalences ranging from 0.35% to 21.98% (Table). Co-infections with 2 other respiratory pathogens, parainfluenza virus type 3 (C_t_ 30) and bocavirus (C_t_ 29), were detected in the mimivirus-positive patient. A sputum smear from this patient was negative for *Mycobacteria tuberculosis*, and other bacteria were not detected.

The mimivirus-positive patient had neonatal respiratory distress syndrome at birth and had been hospitalized 6 times because of reoccurring respiratory tract infections before the episode during which mimivirus was detected. On July 24, 2013, he had a sudden onset of a slight fever (37.8°C), cough, and diarrhea (6–7 bowel movements/day). After he was given supportive treatment, diarrhea improved, while fever and cough were aggravated; onset of larynx asthma was also recorded. He was admitted to the Respiratory Department of Children’s Hospital of Chongqing Medical University on July 28. Physical examination at admission showed lip cyanosis and 3 depression signs. Pulmonary computed tomography after hospitalization showed inflammation of the left upper lung and right lung. Laboratory investigations at admission showed a platelet count of 484 × 10^9^/L, an erythrocyte count of 4.28 × 10^9 ^cells/L, a hemoglobin level of 86 g/L, and a leukocyte count of 8.28 × 10^9^ cells/L with 17% neutrophils and 78% lymphocytes.

The patient was given symptomatic supportive treatment, methylprednisolone sodium succinate, and 5 g of γ-globulin. He was not given any antimicrobial drugs. On August 4, he was discharged from hospital after symptoms had resolved.

In conclusion, our results confirm that mimivirus is an unlikely cause of human respiratory infections in China, as reported in other countries (*5*–*9*). Sporadic detection of mimivirus in 1 child who was born with a compromised respiratory system and had numerous hospitalizations was most likely caused by colonization of the child with this virus during numerous hospitalizations and critical care stays. In addition, parainfluenza virus 3 and bocavirus were detected in the mimivirus-positive child. Because parainfluenza virus 3 causes pneumonia and bocavirus causes infections with respiratory symptoms, particularly in children of his age, these 2 pathogens probably caused the illness in the child.

**Table:** Prevalence of 11 other viruses in 3,696 patients with respiratory diseases tested for infection with mimivirus, China

Virus	No. (%) patients
Influenza virus	307 (8.31)
Respiratory syncytial virus	812 (21.97)
Parainfluenza virus type 1	275 (7.44)
Parainfluenza virus type 2	13 (0.35)
Parainfluenza virus type 3	253 (6.85)
Parainfluenza virus type 4	38 (1.03)
Metapneumovirus	85 (2.30)
Human rhinovirus	543 (14.69)
Human adenoviruses	123 (3.33)
Coronavirus	79 (2.14)
Human bocavirus	179 (4.84)
